# Apparent mineralocorticoid excess with a novel *HSD11B2* variant: longitudinal clinical and biochemical follow-Up

**DOI:** 10.1210/jcemcr/luag083

**Published:** 2026-04-29

**Authors:** Bhanu Teja Nalluri, Neelaveni Kudugunti, Rakesh Kumar Sahay

**Affiliations:** Department of Endocrinology, Osmania Medical College, Hyderabad 500001, India; Department of Endocrinology, Osmania Medical College, Hyderabad 500001, India; Department of Endocrinology, Osmania Medical College, Hyderabad 500001, India

**Keywords:** apparent mineralocorticoid excess, *HSD11B2*, pediatric hypertension, nephrocalcinosis, monogenic hypertension

## Abstract

Apparent mineralocorticoid excess (AME) is a rare autosomal recessive disorder caused by loss-of-function mutations in *HSD11B2*, leading to impaired conversion of cortisol to cortisone and inappropriate mineralocorticoid receptor activation. Early recognition is critical, as untreated disease results in progressive cardiovascular and renal complications. We describe a 2-year-old boy with severe early-onset hypertension, polyuria, polydipsia, abdominal distension, and growth retardation. Biochemistry revealed hypokalemic metabolic alkalosis with suppressed renin and low aldosterone. Renal ultrasonography demonstrated bilateral medullary nephrocalcinosis. Whole-exome sequencing identified a novel homozygous missense mutation in *HSD11B2* (c.478G>A; p.Gly160Ser)—previously reported as a variant of unknown significance. Targeted therapy with spironolactone, a thiazide diuretic, potassium supplementation, and adjunct antihypertensives achieved normalization of blood pressure and potassium levels, resolution of alkalosis, and significant catch-up growth over 6 months, with stable renal function. This case expands the mutational spectrum of *HSD11B2* and demonstrates that early genetic confirmation and tailored therapy can reverse biochemical abnormalities, promote growth, and prevent long-term sequelae. AME should be considered in children with low-renin, low-aldosterone hypertension, particularly in consanguineous populations.

## Introduction

Hypertension in children is uncommon and often indicates an underlying secondary or monogenic cause. Apparent mineralocorticoid excess (AME) is a rare autosomal recessive disorder resulting from mutations in *HSD11B2*, which encodes enzyme 11β-hydroxysteroid dehydrogenase type 2 (11β-HSD2) [1-3]. Under normal physiology, 11β-HSD2 converts cortisol to cortisone, thereby protecting the mineralocorticoid receptor from inappropriate cortisol activation [1, 4]. Deficiency of this enzyme allows cortisol to act as a potent mineralocorticoid, driving sodium retention, potassium loss, metabolic alkalosis, and volume expansion, which manifest clinically as severe low-renin hypertension [1-3].

Children with AME typically present in infancy or early childhood with resistant hypertension, failure to thrive, hypokalemia, and metabolic alkalosis [2, 5]. Nephrocalcinosis and renal impairment may develop due to chronic electrolyte imbalance [2, 5]. More than 50 pathogenic *HSD11B2* variants have been identified, but genotype–phenotype correlations remain incomplete [3, 5]. The clinical picture overlaps with other causes of low-renin hypertension, including Liddle syndrome, congenital adrenal hyperplasia (11β- or 17α-hydroxylase deficiency), deoxycorticosterone-producing tumors, Cushing syndrome, and exogenous mineralocorticoid exposure, making genetic confirmation essential for accurate diagnosis [3, 6].

Management includes mineralocorticoid receptor antagonists (spironolactone or eplerenone), potassium supplementation, dietary sodium restriction, and thiazide diuretics in patients with nephrocalcinosis. Low-dose glucocorticoids may be considered in refractory cases but raise concerns regarding growth suppression in young children [1, 2, 4]. Early recognition and treatment improve long-term outcomes by preventing irreversible cardiovascular and renal damage. Genetic counseling is essential in consanguineous families where recurrence risk is high [2, 3, 7].

## Case presentation

**Figure 1:**
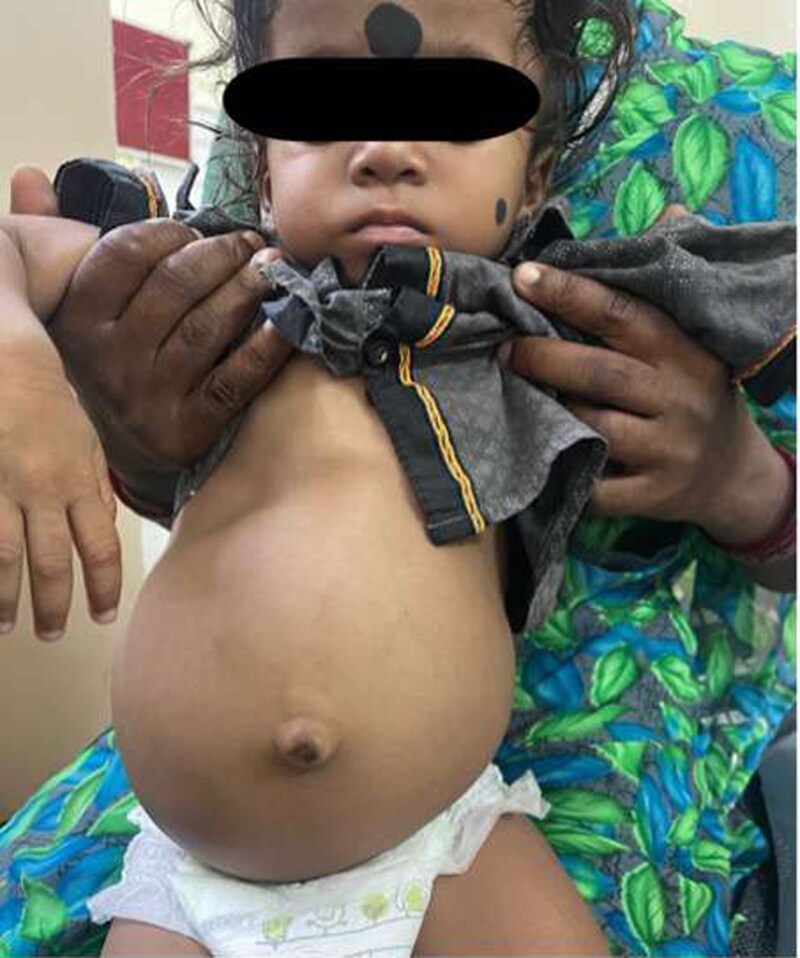
Clinical image of the child showing protruded abdomen.

A 2-year-old boy, the first child of third-degree consanguineous parents, presented with a 5-month history of polyuria, polydipsia, abdominal distension, and poor weight gain as seen in Fig. 1. He was born at 37 weeks following a pregnancy complicated by maternal hypertension, with a birth weight of 1.5 kg (<3rd percentile). Developmental milestones were age appropriate.

On examination, weight (7.5 kg) and height (68 cm) were both below the 3rd percentile. Blood pressure was markedly elevated (150/100 mmHg, >95th percentile for age). There was no edema, cushingoid features, or dysmorphism. Genital examination revealed normal male looking genitalia with no ambiguity and cardiovascular, respiratory, and neurological examinations were unremarkable.

## Diagnostic assessment

Routine blood investigations revealed normal complete blood counts and liver function tests. The estimated glomerular filtration rate (eGFR), calculated using the Schwartz equation, was mildly reduced at 69.7 mL/minute/1.73 m^2^ (SI: 69.7 mL/minute/1.73 m2) (normal: >90 mL/minute/1.73 m2). Electrocardiogram and echocardiography showed no evidence of cardiac hypertrophy. Thyroid function tests were normal, with a thyroid stimulating hormone (TSH) level of 3.41 μIU/mL (normal: 0.5-4.5 μIU/mL).

Venous blood gas analysis demonstrated metabolic alkalosis pH 7.5 (normal: 7.31-7.41 for venous), HCO_3_^−^ of 31 mmol/L (SI: 31 mmol/L) (normal: 22-28 mmol/L). Serum electrolytes showed hypokalemia 2.4 mmol/L (normal: 3.5-5.0 mmol/L) with normal sodium 142 mmol/L (normal: 135-145 mmol/L). Plasma renin activity was suppressed at 0.53 ng/mL/h (SI: 0.53 μg/L/h) (reference range, 1.31-3.95 ng/mL/h [SI: 1.31-3.95 μg/L/h] in the upright posture), and serum aldosterone was low at 0.97 ng/dL (SI: 0.0269 nmol/L) (reference range, [SI: 0.069- nmol/L-1.0875 nmol/L] in the upright posture). Serum cortisol was 12.1 μg/dL (SI: 333.79 nmol/L) (reference range, 6.2-19.4 μg/dL [SI: 171-536 nmol/L]), while serum cortisone was markedly reduced at 1.12 nmol/L (reference range [SI: 6.37-49.02 nmol/L]), resulting in an elevated cortisol-to-cortisone ratio of 298 (reference range: 1.63-5.15). Serum 17-hydroxyprogesterone was 1.2 ng/mL (SI: 3.6 nmol/L) (reference range, <2.0 ng/mL [SI: <6.1 nmol/L]), and serum deoxycorticosterone was 0.40 ng/mL (SI: 1.2 nmol/L) (reference range, 0.46-2.51 ng/mL [SI: 1.4-7.6 nmol/L]).

**Figure 2:**
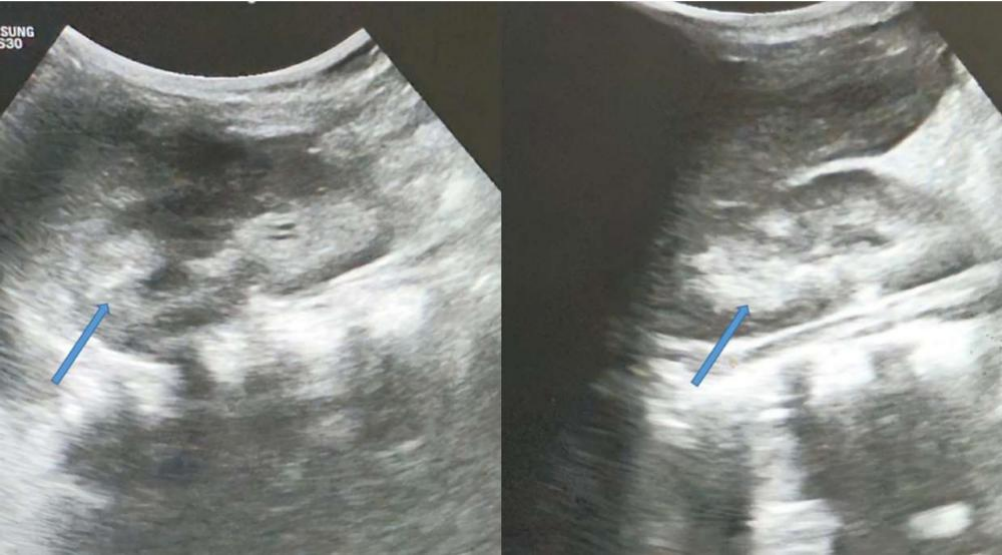
abdominal ultrasound showing bilateral medullary nephrocalcinosis marked by arrows.

Abdominal and pelvic ultrasonography revealed bilateral medullary nephrocalcinosis with grade 1 renal parenchymal changes as seen in Fig. 2. The liver was normal in size and architecture, with no splenomegaly or ascites. Renal Doppler imaging excluded renovascular disease. Urinary calcium excretion was elevated, with a spot urine calcium-to-creatinine ratio of 0.4 (normal: <0.2). Dilated fundus examination revealed no evidence of hypertensive retinopathy.

Whole-exome sequencing identified a novel homozygous missense mutation in *HSD11B2* (c.478G>A; p.Gly160Ser)—previously reported as a variant of unknown significance, predicted to impair 11β-HSD2 function, along with biochemistry and clinical picture confirming the diagnosis of AME.

Several disorders can manifest with low-renin, low-aldosterone hypertension, and each must be carefully excluded before establishing a diagnosis of AME.

Liddle syndrome results from *ENaC* gain-of-function mutations (*SCNN1B/SCNN1G*), leading to sodium retention, hypertension, hypokalemia, and metabolic alkalosis with suppressed aldosterone levels. In the present case, the absence of these mutations and the presence of nephrocalcinosis favored AME as the underlying etiology [1, 2]. Congenital adrenal hyperplasia (CAH) due to 11β-hydroxylase or 17α-hydroxylase deficiency can cause excess deoxycorticosterone (DOC) production, resulting in mineralocorticoid hypertension with low renin and aldosterone [3]. However, the absence of virilization, hypogonadism, or hormonal abnormalities in our patient effectively excluded CAH. Cushing syndrome may cause hypertension secondary to cortisol excess, but typical cushingoid features and biochemical evidence of hypercortisolism were lacking. DOC-producing adrenal tumors, though rare, may produce a similar biochemical profile; however, imaging in our patient revealed no adrenal mass or enlargement. Lastly, exogenous mineralocorticoid exposure or licorice ingestion (containing glycyrrhizic acid, an inhibitor of 11β-HSD2) can mimic AME. A thorough review of dietary and medication history ruled out these possibilities.

Taken together, the absence of hormonal abnormalities, negative imaging, and genetic confirmation of a novel homozygous *HSD11B2* mutation of unknown significance along with biochemistry established AME as the definitive diagnosis.

## Treatment

The child was initially managed with nifedipine, prazosin, and clonidine for blood pressure control. Persistent hypokalemia required oral potassium supplementation. Following genetic confirmation, spironolactone was introduced and titrated up to 15 mg/kg/day, along with dietary sodium restriction. A thiazide diuretic was added to address nephrocalcinosis. Electrolytes and blood pressure were closely monitored. Low-dose corticosteroid therapy was considered but deferred due to concerns regarding growth suppression [1, 3, 5].

## Outcome and follow-up

The child was followed monthly with close monitoring of blood pressure, electrolytes, growth, and antihypertensive requirements. At diagnosis, he had severe hypertension (150/90 mmHg), hypokalemia (2.4 mmol/L), and growth retardation (weight 7.5 kg; height 68 cm) with both height and weight below the 3rd percentile for age. He was treated with spironolactone in combination with nifedipine, prazosin, clonidine, thiazide, and potassium supplementation.

By 2 months, blood pressure improved (90/70 mmHg), potassium levels rose to 3.1 mmol/L, and modest catch-up growth was observed.

At 4 months, the child achieved normotension (80/60 mmHg) on spironolactone (12.5 mg/kg/day) with normalization of potassium (3.7 mmol/L).

By 6 months, blood pressure stabilized at 70/50 mmHg, calculated glomerular filtration rate by Schwartz equation improved to 114 m/minute/1.73 m2 . Serum potassium normalized (5.2 mmol/L), and weight gain accelerated (14 kg). There is elevation of renin levels due to overtreatment warranting dose reduction of spironolactone.

At 7 months, his antihypertensive regimen was simplified to spironolactone and thiazide, with sustained normotension and improved growth trajectory (15 kg (75 percentile for age), 76 cm (less than 3 percentile for age).

**Figure 3:**
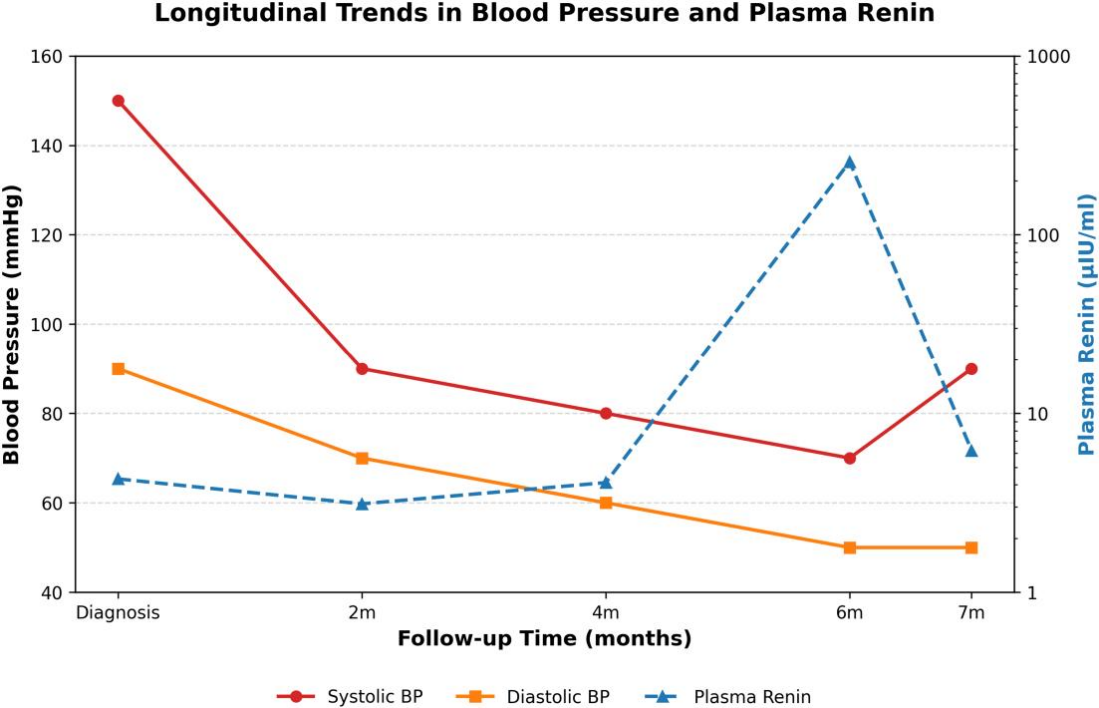
trends of blood pressure and direct renin concentration in the follow up period.

The progressive rise in plasma renin concentrations as depicted in Table 1 and Fig. 3 over follow-up likely reflected successful mineralocorticoid receptor blockade and correction of sodium retention. Renal function remained stable, and metabolic alkalosis resolved.

**Table 1:** Summary of the patient’s longitudinal follow-up, detailing medication regimens, blood pressure measurements, height and weight progression, plasma renin concentrations, and serum potassium levels

Parameter	At diagnosis	2 Months	4 Months	6 Months	7 Months
Blood pressure (95th percentile: 104/57 mmHg)	150/90 mmHg	90/70 mmHg	80/60 mmHg	70/50 mmHg	90/50 mmHg
Direct plasma renin Concentration (normal: 4.4-46.1 μIU/mL) (SI: 4.4-46.1 mIU/L)	4.3 μIU/mL (SI −4.3 mIU/L)	3.12 μIU/mL (SI −3.12 mIU/L)	4.1 μIU/mL (SI −4.1 mIU/L)	256 μIU/mL* (SI −256 mIU/L)	6.2 μIU/mL (SI −6.2 mIU/L)
Serum aldosterone (SI normal: 0.84-2.8 nmol/L upright)	0.97 nmol/L	NA	NA	NA	NA
Serum potassium (SI normal: 3.5-5.0 mmol/L)	2.4 mmol/L	3.1 mmol/L	3.7 mmol/L	5.2 mmol/L	4.3 mmol/L
Height	68 cm	69 cm	72 cm	75 cm	76 cm
Weight	7.5 kg	8 kg	11 kg	14 kg	15 kg
Antihypertensives at admission	Spironolactone 3 mg/kg, Nifedipine 2 mg/kg/day, Prazosin 2.5 mg OD (0.33 mg/kg/day), Clonidine 15 mcg/kg/day, Thiazide 12.5 mg/day + K + supplementation	Spironolactone 8 mg/kg/day, Nifedipine 2 mg/kg/day, Clonidine 15 mg/kg/day, Thiazide 12.5 mg OD + K + supplementation	Spironolactone 12.5 mg/kg/day, Nifedipine 2 mg/kg/day, Thiazide 12.5 mg OD	Spironolactone 14 mg/kg/day, Nifedipine 1 mg/kg/day, Thiazide 12.5 mg OD	Spironolactone 10 mg/kg/day, Thiazide 12.5 mg OD
Antihypertensives at discharge	Spironolactone 8 mg/kg/day, Nifedipine 2 mg/kg/day, Clonidine 15 mg/kg/day, Thiazide 12.5 mg OD + K + supplementation	Spironolactone 12.5 mg/kg/day, Nifedipine 2 mg/kg/day, Thiazide 12.5 mg OD	Spironolactone 14 mg/kg/day, Nifedipine 1 mg/kg/day, Thiazide 12.5 mg OD	Spironolactone 10 mg/kg/day, Thiazide 12.5 mg OD	Spironolactone 10 mg/kg/day, Thiazide 12.5 mg OD

OD, once daily; K+, potassium supplementation.

*Renin elevation due to overtreatment with spironolactone, warranting dose reduction.

## Discussion

AME is a rare, recessively inherited cause of severe pediatric hypertension due to impaired activity of 11β-hydroxysteroid dehydrogenase type 2 (*HSD11B2*) [1]. The resultant failure to inactivate cortisol permits inappropriate mineralocorticoid receptor activation, driving sodium retention, hypokalemia, metabolic alkalosis, and hypertension [1, 2]. Our patient also had bilateral nephrocalcinosis, a recognized complication attributed to chronic hypercalciuria in this disorder [4].This case is notable for the identification of a novel homozygous missense mutation (c.478G>A; p.Gly160Ser) in *HSD11B2—*previously reported as a variant of unknown significance, predicted to impair enzyme function. Reporting such variants expands the known mutational spectrum of AME and contributes to better understanding of genotype–phenotype relationships, which remain incompletely defined. The longitudinal follow-up data in our case underscores the therapeutic effectiveness of mineralocorticoid receptor antagonism with spironolactone, combined with salt restriction and thiazide therapy [1]. Correction of hypokalemia, resolution of metabolic alkalosis, and restoration of growth were achieved within 6 months. The marked improvement in weight gain (from 7.5 to 15 kg in 7 months) highlights the reversibility of growth failure once metabolic derangements are corrected. The rise in plasma renin concentration during follow-up further supports successful blockade of mineralocorticoid receptor overactivity.

Alternative therapeutic strategies, such as low-dose glucocorticoids to suppress endogenous cortisol, may be considered in refractory cases but must be balanced against risks of growth suppression in young children [2]. In our patient, good control was achieved without glucocorticoid therapy, suggesting that tailored therapy based on biochemical monitoring is feasible [1, 2]. This case reinforces the importance of considering monogenic forms of hypertension in children, particularly in consanguineous populations where autosomal recessive conditions are more prevalent [7]. Early molecular diagnosis not only informs targeted therapy but also enables genetic counseling and anticipatory guidance for families.

In conclusion, AME should be strongly suspected in young children presenting with severe hypertension, hypokalemia, metabolic alkalosis, and suppressed renin–aldosterone axis. Genetic confirmation is essential for accurate diagnosis, guiding therapy, and family counseling [1-3]. Our case describes a novel *HSD11B2* mutation (p.Gly160Ser)—previously reported as a variant of unknown significance with detailed follow-up demonstrating reversal of metabolic abnormalities, normalization of blood pressure, and improved growth with spironolactone-based therapy [1-3]. This report highlights the value of early recognition and longitudinal monitoring in optimizing outcomes and preventing long-term renal and cardiovascular sequelae in children with AME.

## Learning points

- Apparent mineralocorticoid excess (AME) is a rare autosomal recessive disorder causing severe pediatric hypertension due to impaired HSD11B2 activity.
- Identification of a novel homozygous missense variant (c.478G>A; p.Gly160Ser) previously reported as a variant of uncertain significance expands the mutational spectrum of AME.
- Nephrocalcinosis is a recognized complication, likely secondary to chronic hypercalciuria.
- Spironolactone, salt restriction, and thiazide therapy achieved effective biochemical and clinical control, with reversal of growth failure. Regular monitoring with renin levels and serum potassium helps in titration of antihypertensives.
- Early molecular diagnosis informs targeted therapy, facilitates genotype–phenotype correlations, and provides opportunities for genetic counseling, particularly in consanguineous populations.

## Data Availability

Data sharing is not applicable to this article as no datasets were generated or analyzed during the current study.
